# Accidental hypothermia–an update: The content of this review is endorsed by the International Commission for Mountain Emergency Medicine (ICAR MEDCOM)

**DOI:** 10.1186/s13049-016-0303-7

**Published:** 2016-09-15

**Authors:** Peter Paal, Les Gordon, Giacomo Strapazzon, Monika Brodmann Maeder, Gabriel Putzer, Beat Walpoth, Michael Wanscher, Doug Brown, Michael Holzer, Gregor Broessner, Hermann Brugger

**Affiliations:** 1 Department of Anaesthesiology and Critical Care Medicine, Innsbruck University Hospital, Anichstr. 35, 6020 Innsbruck, Austria; 2 Barts Heart Centre, St Bartholomew’s Hospital, West Smithfield, Barts Health NHS Trust, Queen Mary University of London, KGV Building, Office 10, 1st floor, West Smithfield, London, EC1A 7BE UK; 3 International Commission of Mountain Emergency Medicine (ICAR MEDCOM), Kloten, Switzerland; 4 Department of Anaesthesia, University hospitals, Morecambe Bay Trust, Lancaster, UK; 5 Langdale Ambleside Mountain Rescue Team, Ambleside, UK; 6 Institute of Mountain Emergency Medicine, EURAC research, Drususallee 1, Bozen/Bolzano, Italy; 7 Department of Emergency Medicine, Inselspital, Bern University Hospital, Bern, Switzerland; 8 Department of Surgery, Cardiovascular Research, Service of Cardiovascular Surgery, University Hospital Geneva, Geneva, Switzerland; 9 Department of Cardiothoracic Anaesthesia and Intensive Care 4142, Copenhagen University Hospital, Rigshospitalet, Copenhagen, Denmark; 10 Department of Emergency Medicine, University of British Columbia, Vancouver, Canada; 11 Department of Emergency Medicine, Medical University of Vienna, Vienna, Austria; 12 Department of Neurology, Neurologic Intensive Care Unit, Medical University of Innsbruck, Innsbruck, Austria

**Keywords:** Cardiopulmonary bypass, Cardiopulmonary resuscitation, Emergency medicine, Extracorporeal membrane oxygenation, Hypothermia, Resuscitation

## Abstract

**Background:**

This paper provides an up-to-date review of the management and outcome of accidental hypothermia patients with and without cardiac arrest.

**Methods:**

The authors reviewed the relevant literature in their specialist field. Summaries were merged, discussed and approved to produce this narrative review.

**Results:**

The hospital use of minimally-invasive rewarming for non-arrested, otherwise healthy, patients with primary hypothermia and stable vital signs has the potential to substantially decrease morbidity and mortality for these patients. Extracorporeal life support (ECLS) has revolutionised the management of hypothermic cardiac arrest, with survival rates approaching 100 % in some cases. Hypothermic patients with risk factors for imminent cardiac arrest (temperature <28 °C, ventricular arrhythmia, systolic blood pressure <90 mmHg), and those who have already arrested, should be transferred directly to an ECLS-centre. Cardiac arrest patients should receive continuous cardiopulmonary resuscitation (CPR) during transfer. If prolonged transport is required or terrain is difficult, mechanical CPR can be helpful. Delayed or intermittent CPR may be appropriate in hypothermic arrest when continuous CPR is impossible. Modern post-resuscitation care should be implemented following hypothermic arrest. Structured protocols should be in place to optimise pre-hospital triage, transport and treatment as well as in-hospital management, including detailed criteria and protocols for the use of ECLS and post-resuscitation care.

**Conclusions:**

Based on new evidence, additional clinical experience and clearer management guidelines and documentation, the treatment of accidental hypothermia has been refined. ECLS has substantially improved survival and is the treatment of choice in the patient with unstable circulation or cardiac arrest.

## Background

The management of accidental hypothermia has made substantial progress over the last two decades and hypothermic cardiac arrest (CA) patients who often do not survive with traditional rewarming methods (e.g. dialysis, pleural lavage) have become increasingly salvageable with extracorporeal life support (ECLS). The aim of this review is to consider the substantial advances made in the last decade in the management and outcome of accidental hypothermia patients with and without CA. Based on new evidence, additional clinical experience and clearer management guidelines and documentation, the treatment of accidental hypothermia has been refined. ECLS has substantially improved survival and is the treatment of choice in the patient with unstable circulation or CA.

## Accidental hypothermia: an update- part 1 definitions, diagnosis, prehospital management and triage

### Introduction

The management of accidental hypothermia has made substantial progress over the last two decades and hypothermic CA patients who often do not survive with traditional rewarming methods (e.g. dialysis, pleural lavage) have become increasingly salvageable with extracorporeal life support (ECLS) [1–8]. New recommendations regarding delayed or intermittent CPR may facilitate patient transport [9].

**Fig. 1:**
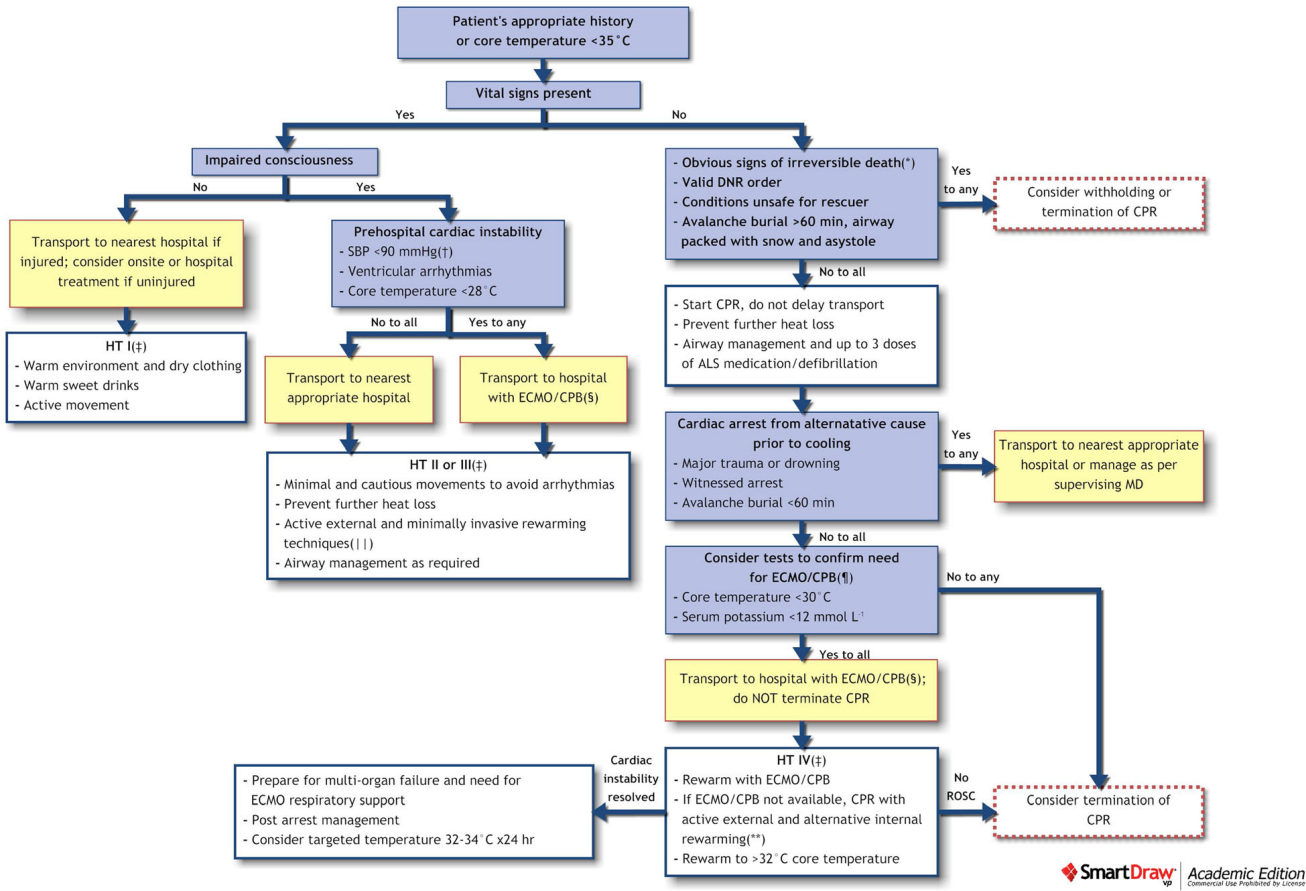
Management in Accidental Hypothermia. (*) Decapitation; truncal transection; whole body decomposed or whole body frozen solid (chest wall not compressible) [128]. (†) SBP <90 mmHg is a reasonable prehospital estimate of cardiac instability but for in-hospital decisions, the minimum sufficient circulation for a deeply hypothermic patient (e.g., <28 °C) has not been defined. (‡) Swiss staging of accidental hypothermia [73], see also Table 1. (§) In remote areas, transport decisions should balance the risk of increased transport time with the potential benefit of treatment in an ECLS centre. (||) Warm environment, chemical, electrical, or forced air heating packs or blankets, and warm IV fluids (38–42 °C). In case of cardiac instability refractory to medical management, consider rewarming with ECLS. (¶) If the decision is made to stop at an intermediate hospital to measure serum potassium, a hospital en route towards the ECLS centre should be chosen. (**) See Table 3. CPR denotes cardiopulmonary resuscitation, DNR do-not-resuscitate, ECLS extracorporeal life support, HT hypothermia, MD medical doctor, ROSC return of spontaneous circulation, SBP systolic blood pressure

Although some pathophysiological mechanisms are similar, accidental hypothermia should neither be compared to induced hypothermia (as used in deep hypothermic circulatory arrest (DHCA) for cardiovascular surgery) nor to therapeutic hypothermia (i.e. in targeted temperature management as part of a post-resuscitation care bundle): i) accidental hypothermia happens unexpectedly and is uncontrolled; ii) it is often associated with exposure to cold environments and/or secondary to impaired thermoregulation e.g. alcohol, drug ingestion, trauma, extremes of age or co-morbid illness [10]. The elderly are at increased risk due to decreased physiologic reserve, chronic disease and medications that impair compensatory responses. The current lowest temperatures from which successful resuscitation and rewarming have been achieved are 13.7 °C [11] for accidental and 9 °C [12] for induced hypothermia. Successful resuscitation at even lower temperatures may be possible. This article gives a state-of-the-art review on accidental hypothermia management. An algorithm is provided in Fig. 1.

### Methods

A working panel of doctors with a special interest in accidental hypothermia, including speakers at the IV^th^ International Symposium on Hypothermia in Bozen/Bolzano (2014), addressed the current management of accidental hypothermia. Each doctor reviewed with individualized Pubmed searches the relevant literature in their field of expertise. Summaries were merged into this narrative review. Following discussion and approval, 279 relevant articles were included.

### Cooling: implications for drowning and avalanche rescue

In accidental hypothermia, cooling rates depend on many factors including cold acclimatisation, body size, age, insulation (clothing and subcutaneous fat), ability to shiver, body movement, temperature gradient, the amount of body surrounded by a cold medium (air, snow or water) and local conditions that increase cooling e.g. wind speed, rough water [13, 14]. The crucial factor in all hypothermia cases is whether critical brain hypoxia occurs before protective brain-cooling takes place [15–18].

In water incidents, management is as for hypothermia on dry land, including careful movement to avoid precipitating a life-threatening arrhythmia [19] and keeping the casualty in a horizontal position when pulled from the water to minimise the likelihood of rescue collapse [20]. These issues are discussed in greater detail later in this review. If an immersion victim (head out) is able to cling to floating wreckage or the edge of an ice sheet, they will eventually become hypothermic, although it may take up to one hour for this to become a life-threatening problem [21–24].

Submersion (head under water) duration is a direct measure of anoxic injury [8, 25] so that 2.5–5 min predicts a good outcome [8, 26–28], >10 min is associated with poor outcome [8, 26, 29, 30] and there is almost no chance of survival >25–30 min [8, 16, 26, 31, 32]. Although hypothermia may reflect a prolonged submersion time and poor prognosis [33], early hypothermia is an important reason why survival without neurologic damage is possible [34]. Instances of miraculous survival with good recovery after submersion have occurred, mainly in small children submersed in icy water who rapidly became hypothermic prior to hypoxia (Table 2) [8, 16, 35–41], but also in adults [19, 42–44]. There are several reasons for improved immediate survival in children after submersion but from a hypothermia perspective, they cool more rapidly than adults during cold water submersion (<6 °C) [21, 45], especially infants, who have an inefficient shivering mechanism [46]. Children have less subcutaneous fat, which at any age leads to faster cooling [13, 19, 47], and a higher surface area to body weight ratio [45]. Aspiration of very cold water into a child’s small body is believed to immediately cool the heart and blood in the carotid arteries and therefore the brain [48–50]. Finally, a small child-sized head will cool faster by conduction than a large adult-sized head [48]. Case reports repeatedly demonstrate a poor outcome after drowning, even after ECLS for rewarming [51–55] and use of targeted temperature management [56]. In drowning, there are currently no methods to reliably predict early on who will survive or the likely long-term neurocognitive outcome, so in the absence of obvious indications of death e.g. rigor mortis [32], published guidance urges aggressive out-of-hospital and in-hospital treatment in all cases [16, 35], particularly in young hypothermic victims [57]. Immediate full CPR (not compression-only without ventilation) [58] after drowning [59, 60] and the early arrival of EMS (<9 min) are associated with increased survival to hospital admission and survival to one month [8, 61–63]. However, outcomes are extremely poor if ALS takes >30 min to achieve ROSC, even with hypothermia [31]. Retrospective analysis of a large number of drowning cases has produced a useful algorithm based on clinical signs and submersion duration to guide management and grade patients according to survival rate [32, 64, 65]. Understanding the implications of submersion duration on outcome is useful where decisions to rescue and care for a casualty pose a risk to the rescue services. This may contribute to the decision to change from rescue to body retrieval [8, 25]. Finally, although water temperature alone does not affect long-term outcome [8, 26, 28, 29], recent guidance for Search & Rescue teams has suggested that for victims who are continuously submerged (i.e. not in a vehicle where there might be an air pocket), if water temperature is warmer than 6 °C, survival/resuscitation is extremely unlikely if submerged >30 min; if water temperature is ≤6 °C, survival/resuscitation is extremely unlikely if submerged >90 min [34].

The cooling rates in snow in avalanche victims vary substantially. Although the fastest reported cooling rate in an avalanche victim is 9.4 °C/h [66], the accuracy of this figure has been questioned because some cooling would be expected to have occurred after extrication [67]. However, another case of 9 °C/h suggests that some patients cool very rapidly [68]. In avalanche burial, survival decreases dramatically after 35 min [17]. Beyond this inflection point, survival is only possible if the airway is patent and oxygen support from the surrounding snow is sufficient. There are some rare extreme examples of avalanche survival [17, 69]. However, if an avalanche victim is found in cardiac arrest, the outcome is poor, even after ECLS for rewarming [1, 4, 70]. An algorithm is available for avalanche rescue [71]. Current guidance is that victims are unlikely to survive if buried >60 min (or initial core temperature is <30 °C) and in cardiac arrest with an obstructed airway on extrication, or buried and in cardiac arrest on extrication with an initial serum potassium > 8 mmol L^−1^ [8].

The wider aspects of body cooling, avalanche rescue, drowning and resuscitation go beyond the remit of this review and are covered extensively elsewhere [17, 72]. In all cases, rescuer safety is paramount [17, 25].

### Staging

Accidental hypothermia is staged by core-temperature measurement and clinical findings. A classification based on the Swiss system (Hypothermia I–IV; HT I–IV) [73] provides useful guidance in the prehospital situation (Table 1) using level of consciousness, vital signs, and core temperature (if available) to indicate hypothermia severity. A fifth stage (Hypothermia V) may be used to denote death due to irreversible hypothermia [74]. The multitude of patient factors (e.g. age, gender, body composition, physical fitness, race, comorbidities, intoxication, multiple trauma and head injuries) [5] means that clinical findings do not consistently correlate with core-temperature [75]. Consequently, CA may occur just below ≈ 32^°^C or vital signs can be present into upper teens [76]. A staging system is a valuable clinical tool to facilitate triage and emergency treatment. However, definitive assessment of the severity of hypothermia requires accurate core temperature measurement.

**Table 1:** Staging of accidental hypothermia [73]

Stage	Clinical findings	Core temperature (°C) (if available)
Hypothermia I (mild)	Conscious, shivering^a^	35–32 °C
Hypothermia II (moderate)	Impaired consciousness^a^; may or may not be shivering	<32–28 °C
Hypothermia III (severe)	Unconscious^a^; vital signs present	<28 °C
Hypothermia IV (severe)	Apparent death; Vital signs absent	Variable^b^

^a^Shivering and consciousness may be impaired by comorbid illness (i.e. trauma, CNS pathology, toxic ingestion, etc.) or drugs (i.e. sedatives, muscle relaxants, narcotics etc.) independent of core temperature

The lowest temperature from which successful resuscitation and rewarming has been achieved is currently 13.7 °C [11] for accidental hypothermia and 9 °C for induced hypothermia [12]. This does not preclude resuscitation attempts at even lower temperatures if clinical judgment suggests the possibility of successful resuscitation

^b^The risk of cardiac arrest increases below 32 °C, but as it is unlikely to be due solely to hypothermia until the temperature is <28 °C, alternative causes should be considered. Some patients still have vital signs <24 °C and the lowest reported temperature of a patient with vital signs is 17 °C [232, 233]

### Temperature measurement

Core-temperature measurement is essential for diagnosing hypothermia and assessing severity. The practical principles are identical for pre- and in-hospital use. Temperature measurement sites in order of decreasing invasiveness include pulmonary artery, oesophagus, bladder, rectum, tympanic membrane, oral cavity, and skin. Pulmonary artery temperature reflects central blood temperature and is the gold standard [77], but is too invasive for routine use, and it may precipitate an arrhythmia. Oesophageal temperature closely correlates with pulmonary artery temperature [77] if the probe is placed in the lower third of the oesophagus [78], and is standard for patients with a secured upper airway [77, 79]. Pre-hospital measurement in the bladder or rectum is impractical. The patient has to be partially undressed, rectal probes must be inserted to a depth of ≥15 cm, values of both lag behind core during rapid cooling and rewarming [80, 81] and may be falsely elevated if warmed peritoneal lavage is used [79]. For in-hospital measurement of core temperature, inserting a urinary catheter with a temperature probe is a practical method in hypothermic spontaneously-breathing patients, as it allows for simultaneous core temperature measurement and monitoring of urine output.

Epitympanic temperature is reliable in patients with spontaneous circulation, but may give falsely low values with unstable or absent circulation [10]. In a cold environment, epitympanic probes are only reliable after stabilising for a few minutes [82, 83], if the ear canal is unobstructed and insulated from cold air [83–85]. Tympanic temperature measurement should be by thermistor or thermocouple probes placed near the tympanic membrane, but currently there are no epitympanic thermometers with an insulation barrier available for out-of-hospital use. Infrared tympanic thermometers are inaccurate in a cold environment [81, 86, 87], in the presence of hypothermia [88, 89], if the head is cold [90, 91] or hypothermia onset is rapid [92]. Oral temperature is accurate in normothermia [93], but not in the cold [94] and is affected by other factors e.g. head and face temperature [90, 91, 95] making it unreliable. Skin and exposed sites do not accurately reflect core temperature due to poor peripheral circulation and should not be used [94]. Non-invasive flux-method devices have been developed [96] but validation studies in hypothermic patients exposed to a cold environment are required.

### Prehospital insulation, rewarming, rescue collapse and afterdrop

Shivering and active movement are very efficient mechanisms of heat production and are effective rewarming strategies for patients who are fully conscious and able to move [97]. At rest, shivering increases heat production up to five times but also increases oxygen-requirements [98]. Shivering ceases when energy stores are depleted (within hours), core-temperature drops below an individual’s threshold (Table 1), consciousness is impaired, or sometimes during exogenous skin heating [99]. Multiple trauma, other co-morbidities, intoxication, analgesia or sedation [100] may hasten cooling by impairing central and peripheral thermoregulation (i.e. shivering and vasoconstriction). When shivering ceases (HT II–IV), minimal rewarming occurs [101, 102] and in the absence of active rewarming, cooling rate increases [103].

A warm, sweet, non-alcoholic drink will not provide enough heat to rewarm a patient with HT I but will supply carbohydrate to fuel continued shivering [79, 104, 105]. Heat redistribution within the body can cause a continued fall in core temperature after removal from the cold stress–a phenomenon called afterdrop that may also occur during rewarming. Experimental studies [106–108], have demonstrated a ~0.5–1 °C afterdrop during minimally invasive rewarming and slightly more during exercise-assisted rewarming. However, patients often rewarm faster in the exercise group and no adverse outcomes have ever been observed in either group. Some experts argue that hypothermic patients should neither stand nor walk for 30 min after rescue-care commences due to concerns that exercise may exacerbate afterdrop and cause harm [97]. In practice, patients who are awake and alert should not be prevented from mobilising if this will help the rescue [109, 110].

Emergency medical services should have protocols and equipment for managing hypothermic patients [111, 112]. The optimal prehospital transport and rewarming strategies are unknown but some human studies have compared insulation methods [113–116]. Lack of adequate insulation and heat during transport allows continued cooling, thereby increasing the risk of CA. Insulation from cold, wet, and wind as soon as possible is essential, particularly when removing an avalanche victim, as the cooling rate after extrication is faster than during burial [67]. Experimental evidence and experience indicate that in a patient with spontaneous circulation, pre-hospital patient packaging should include a sealed impermeable vapour barrier [114, 117] (if the patient is wet) excluding the face [118, 119], an external heat source, dry insulation (the thicker, the better), and a wind barrier that also reflects heat [114]. Bubble wrap is light-weight and water-resistant so can form part of a packaging system. However, it is available in different thicknesses with different insulation properties and is of limited use on its own [114, 120–122]. Removing wet clothes increases patient comfort but results in rapid cooling if done in a cold or windy environment [74, 115, 119] and is unnecessary if a vapour barrier is used [115, 119]. External heat (e.g. chemical heat-packs, warm water bottles or forced air blankets) should be applied to the head [123], torso and neck areas [117] during transport. Heat must never be applied directly to skin because of the risk of burns [117]. Ideally the outer packaging layer will provide a wind barrier to minimise convective losses and a heat reflector will minimise radiation losses. With short transports (e.g. <60 min), active warming may be only minimally helpful and expensive. For longer journeys (e.g. >60 min), active warming should be used for HT I–III patients. Patients in CA (HT IV) being transported for ECLS-rewarming should ideally have their core-temperature monitored and heat delivery should be titrated to maintain the core body temperature. Prehospital rewarming or cooling of HT IV during transport should be avoided.

Careful handling and avoidance of rough movements are mandatory, especially in patients who have not arrested. Patients should ideally be transported horizontally by stretcher to decrease position-associated changes in venous return, which may increase the risk of post-rescue CA (rescue collapse), particularly if removed from cold water [20].

### Triage and prognostication of accidental hypothermic patients in cardiac arrest

Brain oxygen-consumption decreases by ~6 % per 1 °C fall in core temperature [8] and reaches 16 % at 15 °C [124] compared with normothermia. This improves the brain’s tolerance for low- or no blood-flow states. At 18 °C the brain tolerates CA for up to 10 times longer than at 37 °C [8].

Many factors affect outcome from hypothermic arrest: (1) hypoxia (the most important single factor) [125]; (2) patient considerations (e.g. age, co-morbidities, trauma); (3) speed of cooling; (4) environment (air, water, snow); (5) CA features (body temperature; whether hypoxia preceded arrest; delay before instituting CPR, and CPR quality); (6) rescue considerations (e.g. adequate training to manage a low flow or no blood-flow state; speed of hospital transfer); (7) proximity of appropriate hospital facilities; (8) whether hospital staff appreciate the special requirements of these cases. The best chance of full recovery from hypothermic CA occurs in the previously-well patient with witnessed CA, in which continuous CPR is implemented immediately, timely ECLS commences and appropriate critical care support is available after return of spontaneous circulation (ROSC). Trying to estimate the survivable duration of CA without CPR is fraught with uncertainty, but knowing the maximum times recommended for cardiovascular surgery when conditions are optimal (~25 min for adults >60 years or ~40 min for neonates undergoing DHCA at ~18 °C) may provide a starting point [9].

Avalanche victims who survived neurologically intact (i.e. Cerebral Performance Category (CPC) 1–2) [126] were found in ventricular fibrillation (VF) or pulseless electrical activity (PEA) [2, 4, 127]. Survival chances are very low in buried avalanche victims with unwitnessed asystole at extrication [2, 4] because hypoxia has generally preceded CA leading to irreversible brain damage and death within minutes.

Clinicians have looked for laboratory markers to guide management decisions in difficult cases, but accurately predicting outcome in accidental hypothermia is notoriously difficult. Consequently, the guiding principle is that in the absence of signs incompatible with life, “No one is dead until warm and dead”, regardless of body temperature [8, 128]. The decision to withhold ECLS-rewarming is usually best-taken at the receiving hospital. Reasons may include death by hypoxia before hypothermia, K^+^ > 12mmolL^-1^, and any condition that is unlikely to be survivable in its own right e.g. major trauma, traumatic brain injury, intracerebral haemorrhage or end stage disease [8, 10]. Although no patient with hyperkalaemia >12 mmol/L has ever been successfully resuscitated, many patients with a normal potassium also do not survive so the utility of serum potassium for clinical decision-making is limited. A retrospective review of avalanche victims who underwent computer tomographic (CT) scanning in hospital suggests that admission serum potassium concentration was higher in patients with CT-verified brain anoxia compared to patients with a normal CT scan [129]. Current European Resuscitation Council (ERC) guidelines [8] recommend a potassium threshold of 8 mmol L^-1^ for avalanche victims and 12 mmol L^-1^ for other causes of accidental hypothermia [8] because one adult avalanche victim with a potassium of 6.4 mmol L^-1^ [130] and a child cooling outdoors (14.2 °C) with a potassium of 11.7 mmol L^-1^ [131] both survived neurologically intact (Table 2).

**Table 2:** The most extreme reported accidental hypothermia cases

Longest no flow time	42-year-old male, found in crevasse, 7 m under snow, no vital signs, CPR started only after 70 min in hospital when patient was asystolic, 19 °C core temperature, ECLS rewarming, full recovery [211].
Longest manual CPR	42-year-old male, found outdoors. Developed asystole just after discovery, CPR started, 23.2 °C, 6 h and 30 min CPR. Rewarmed with non-ECLS methods until ROSC. Full recovery [143].
Longest mechanical CPR	42-year-old female, found unconscious in her apartment. VF arrest during evacuation to hospital. Manual CPR started and this was changed to mechanical CPR on arrival at hospital. Minimal temperature 24 °C. 80 min mechanical CPR while the patient was rewarmed noninvasively [153].
Longest total resuscitation	65-year-old female went missing and was found on a snow-covered riverbank. Initially 28 °C (rectal) but dropped to 20.8 °C. Asystole. Resuscitation was CPR (4 h 48 m) and ECLS (3 h 52 m). Total resuscitation time was 8 h 40 min [142].
Lowest survived body core temperature	29-year-old female, fell into water fall gully, flooded by icy water but able to breathe. Lifeless for approx. 45 min, CPR started after rescue, at hospital admission 13.7 °C and K+ of 4.3 mmol L^-1^, ECLS rewarming, full recovery [11].
Longest persisting VF	42-year-old male, found outdoor, CPR started, repeated shocks, hospital transfer, 22 °C, ECLS rewarming started at 130 min CPR and after 38 shocks, successful shock at 30 °C, full recovery [234]. 25-year-old female, buried by and avalanche in Tatra mountains, Poland. Witnessed VF cardiac arrest (17.0 °C) after extrication, 3 unsuccessful shocks. CPR until ECMO rewarming (6 h, 45 min), and successful 4^th^ shock at 24.8 °C. Full recovery [235].
Longest intermittent CPR	57-year-old female, witnessed cardiac arrest in French Alps at 2000 m altitude in a snowstorm; transport distance to EMS vehicle of 1.1 km, 122 m difference in height; 1 min CPR alternating with 1 min walking for 25 min, 5 h CPR, ECLS rewarming, full recovery [69].
Longest submersion	2.5-year-old, submersion in cold water for at least 66 min, 19 °C, ECLS rewarming, full recovery [38]. 7-year-old child, submersion in icy water for at least 83 min, CPR for 64 min, 13.8 °C, K+ 11.3 mmol L^-1^, ECLS rewarming, full recovery [212].
Longest survival in an avalanche	Female, core temperature <32 °C, when found somnolent, disorientated. 1st- 2nd degree frost bites on hand and feet, no injuries, 43 h and 45 min [236, 237].
Longest time in an avalanche indoor	Thirteen days entrapped in a house which in part collapsed after being hit by an avalanche, Heiligenblut, Austria [238].
Lowest temperature with vital signs	Male age 3 years. ECG showed very irregular rhythm 8–10/min. Rectal temperature recorded about 20 min after arrival at the hospital was 17 °C [232]. Female age 37 years. Rectal temperature 17.2 °C. ECG showed atrial fibrillation 28–40/min with PVCs [233].
Highest survived potassium in an avalanche victim	Avalanche victim, 6.4 mmol L^-1^, survived; core temperature and neurological outcome are not reported [130].
Highest survived potassium in an adult	34 year old female, 20 °C, cold environment exposure, asystole, 7.9 mmol L^-1^, ECLS rewarming, survived. Neurologic outcome not reported [239].
Highest potassium in an accidentally hypothermic patient	7 -year-old and, cold water submersion, 11.3 mmol L^-1^ [212], and 31 month old child, cold water submersion, 11.7 mmol L^-1^ [131].
Longest time in a crevasse	27 -year-old male, 8 days, good outcome, no temperature or other specific details reported [240] 70 year male, moderate fractures of skull, vertebral column, pelvis, and femur, 6 days, 33.5 °C, cold injuries to toes, otherwise good outcome [241].
Largest number of simultaneous cases of accidental hypothermia with cardiac arrest	15 healthy subjects age 15–45 years were immersed in 2 °C salt water. Seven victims were recovered in circulatory arrest with a median temperature of 18.4 °C. They were rewarmed with ECMO and were subsequently evaluated with advanced neuroradiological and functional testing. All were successfully resuscitated [41].

### Oxygenation, anaesthesia induction and airway management

Indications to secure the airway do not differ from recommendations in normothermic patients [8]. Intubation may provoke ventricular fibrillation (VF) in severe hypothermia [132, 133] but the risk is small [8, 134, 135]. There is little published data about anaesthesia in these patients, but the likely effects can be anticipated by extrapolating from studies done on animals, and in patients with induced hypothermia for medical treatment. Most intravenous anaesthetic induction agents cause cardiovascular depression so doses should be small. Ketamine may be safe in pre-existing hypothermia [136], but the sympathomimetic effects could theoretically cause problems for an irritable hypothermic heart [99]. If succinylcholine is used for intubation, the potential for it to increase serum potassium should be considered [137]. Neuromuscular transmission decreases during hypothermia, even in the absence of muscle relaxants [138] and studies performed in animals and humans during hypothermic cardiopulmonary bypass (CPB) have indicated that hypothermia <32 °C increases sensitivity to non-depolarising muscle relaxants [139]. Hypothermia reduces the systemic clearance of CYP450-metabolised drugs (including propofol and ketamine) by an amount proportional to the fall in body temperature, increasing the likelihood of unanticipated toxicity [140, 141].

During anaesthesia induction and intubation, continuous ECG monitoring, CPR-preparedness and placement of defibrillation-pads are recommended. Normocapnia should be maintained during airway management and thereafter [135], because hypercapnia and hypocapnia can induce arrhythmias [79]. Inspired oxygen can be titrated against pulse oximetry (if peripheral perfusion allows) or blood gas analysis (if available), as normoxia is believed to protect from arrhythmia [135]. During CPR, ventilation should be provided as in normothermic CA patients.

### Cardiopulmonary resuscitation

**Fig. 2:**
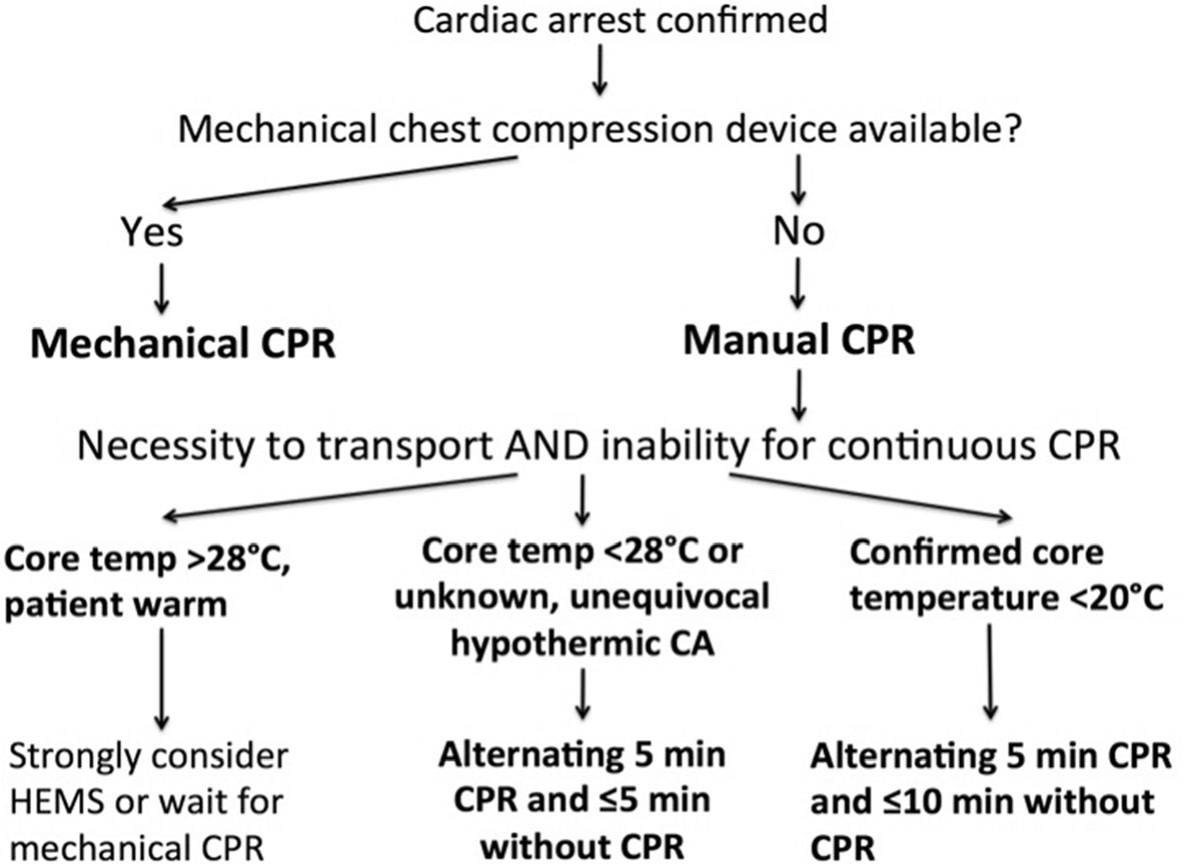
Delayed and intermittent CPR in in hypothermic patients when continuous CPR is not possible during difficult rescue missions [9]

Patients in hypothermic arrest often need prolonged CPR [3, 142–144]. High-quality CPR is the key to best outcome. During technically-challenging evacuation from difficult terrain, manual CPR may be impaired or impossible [145–147]. Mechanical chest compression devices can deliver >50 % of baseline cerebral blood flow in normothermic animals [148], and therefore are likely to provide sufficient oxygen delivery to vital organs in deeply hypothermic patients. They are of value during transport, and to maintain CPR whilst ECLS is being instituted [43, 149–161]. When mechanical CPR is not available and manual CPR is not feasible, intermittent >CPR has been suggested, based on three cases and extrapolation of clinical data from cardiovascular surgery under DHCA (Fig. 2) [9].

Continuous monitoring of CPR efforts to optimize cerebral blood flow is desirable. End-tidal CO_2_ (ETCO_2_) is commonly used as a marker for CPR quality [162, 163] but is not a surrogate for cerebral oxygenation, and it is unclear how to interpret the readings in hypothermia when CO_2_ production is reduced. Near-infrared spectroscopy (NIRS) is increasingly used to monitor regional cerebral oxygen saturation (rSO_2_) during CPR. NIRS may be used to predict ROSC [164, 165] and possibly favourable neurological outcome [166], though current evidence is weak [167, 168]. In an experimental hypothermic CA model, rSO_2_ values closely correlated with invasive parameters of cerebral oxygenation such as cerebral perfusion pressure and cerebral venous oxygen saturation during chest compressions [169]. To date, there is too little clinical experience to recommend NIRS for routine use in hypothermic CA [170].

During normothermic CA, vasopressors are administered to augment coronary perfusion pressure and myocardial blood flow with the aim of increasing ROSC and survival to neurologically-intact hospital discharge [171]. In hypo- (≤28 °C) and normothermic animal [171–174] and normothermic human studies, vasopressor administration was associated with improved ROSC [175–177], but not an increase in survival-to-hospital discharge or better neurological outcome [175, 178–180]. Larger doses of adrenaline may be associated with unfavourable neurological outcome [181], and the peripheral vasoconstriction may worsen concurrent frostbite [17, 182, 183]. Because the adrenaline question has not been resolved, international guidance differs. The ERC 2015 guidelines recommend withholding adrenaline administration in hypothermic CA (HT IV) and limiting defibrillation to three attempts until the core temperature is >30 °C [8]. By contrast, the American Heart Association guidelines allow further defibrillation attempts concurrent with rewarming strategies and state that it may be reasonable to consider adrenaline administration during CA according to the standard ALS algorithm [184, 185]. In HT III, adrenaline has reduced effectiveness and accumulates, although whether this leads to overdose after rewarming is not clear [186]. In HT IV, vasopressors should probably be restricted to weaning from ECLS. Although a few case reports have shown sustained ROSC after defibrillation at <28 °C [41, 131, 144, 187–192], most attempts are unsuccessful. A maximum of three defibrillations appears reasonable <30 °C and then waiting for rewarming to ≥30° before further attempts [8]. Given the on-going controversies, it is acceptable to use either approach until further data becomes available.

The benefit of antiarrhythmic drugs in hypothermic CA is unclear. Many arrhythmias (e.g. bradycardia, atrioventricular blocks, atrial fibrillation, nodal rhythms and QRS prolongation with or without Osborn J-waves) are considered benign in accidental hypothermia, usually regress with patient rewarming and do not require further treatment provided the perfusion is deemed adequate. Pacing may be ineffective in these patients and is not commonly recommended [8].

### Dispatching and transport decisions

Patients with haemodynamic instability or CA [10] should ideally be rapidly transported directly to an ECLS hospital as outcome is better, with survival rates of up to 100 %, compared with other rewarming techniques [41]. The ECLS centre must be contacted early to allow time to organise the team and operating facilities before the patient arrives [160]. There are many examples proving that in hypothermic arrest, good neurological recovery is possible following many hours of CPR, even with prolonged transport [1, 3, 132, 142–144, 187, 190, 193, 194]. If rewarming in an ECLS centre is impossible, it may be attempted in the nearest hospital if a dedicated team is available [195]. If available, an emergency team with portable ECMO may be dispatched to a peripheral hospital [41].

### Conclusions

Cooling rates may vary widely according to the individual situation. Insulation, hypothermia staging, and triage to the appropriate hospital are key. Hypothermic patients with risk factors for imminent cardiac arrest (temperature <28 °C, ventricular arrhythmia, systolic blood pressure <90 mmHg), and those who have already arrested, should be transferred directly to an ECLS-centre. Cardiac arrest patients should receive continuous cardiopulmonary resuscitation (CPR) during transfer. Delayed or intermittent CPR may be appropriate in hypothermic arrest when continuous CPR is impossible. If prolonged transport is required or terrain is difficult, mechanical CPR can be helpful. Outcome is best if hypothermic cardiac arrest is witnessed, high quality CPR performed continuously until ECLS rewarming is started.

## Accidental hypothermia: an update- part 2 in-hospital management

### In-hospital management of hypothermic patients

Rewarming methods can be classified as passive (protection from further heat loss whilst the patient raises their own body temperature), active external (delivery of heat to the surface of the body) and active internal (delivery of heat to the interior of the body). Common rewarming methods, their effectiveness in the hospital setting, controversies and potential complications are presented in Table 3 [10]. Rewarming methods should be evaluated based on morbidity, mortality, patient comfort and resource-use efficiency. Attempts should not be made to rewarm frostbitten extremities until core temperature is >34 °C [196]. During rewarming, all hypothermic patients will have significant intravenous fluid requirements as vasoconstriction relaxes and cold diuresis-mediated dehydration is reversed. Avoidance of hypovolaemia is also important in the presence of frostbite [197].

**Table 3:** Rewarming techniques in accidental hypothermia

Rewarming technique	Rewarming rate	Notes & controversies	Rewarming complications
PASSIVE REWARMING [79]			
Passive rewarming	0.5–4 °C hr^-1^ (dependent upon patient’s thermoregulatory function and metabolic reserves) [79, 242].	Protect from further heat loss and allow patient to self-rewarm. Minimal controversy for mild hypothermia if the patient is able to self-rewarm.	Negligible in isolated mild hypothermia. For colder patients and those with secondary hypothermia or comorbid illness, there may be morbidity associated with a prolonged rewarming process if the patient has poor tolerance for the hypothermia-induced organ dysfunction (i.e. hypotension, coagulopathy, arrhythmias, impaired cellular function etc.).
Passive rewarming with active movement	1–5 °C hr^-1^	Exercise has been shown to increase afterdrop in physiology studies from ~0.3 °C in controls to ~1 °C in exercised subjects, however the exercised subjects rewarmed more quickly [243].	No reported complications. Some authors highlight the theoretical risk that the slightly increased afterdrop could contribute to morbidity and mortality. No adverse events were noted [243].
ACTIVE EXTERNAL REWARMING			
Active rewarming e.g. forced air surface [244] Arctic Sun® [245–247]	0.5-4 °C hr^-1^	Protect from further heat loss, deliver external heat and (if required) warmed IV fluids. Minimal controversies.	Similar to passive rewarming.
ACTIVE INTERNAL REWARMING			
Bladder lavage	Variable. Adds ~0.5–1 °C hr^-1^	Helpful if rewarming rate is slow. Minimal controversies. Rewarming is intermittent & slow because of small surface area. Poor control of infusate temperature [242, 248, 249].	Negligible unless difficult catheterization.
Gastric lavage	Adds ~0.5–1 °C hr^-1^	Not commonly used due to risk vs. benefit ratio [249].	Potential for aspiration, fluid & electrolyte shifts.
Intravascular catheter rewarming e.g. Icy® catheter (CoolGuard®) [76, 250–252] Quattro® [253] Cool Line® [254] Innercool® [255]	Device specific (adds ~0.5–2.5 °C hr^-1^)	Uncertain indication for use, potential for benefit exists in colder and sicker co-morbid patients with stable circulation.	Potential for haemorrhage or thrombosis, potentially worsening hypotension in unstable patients.
Thoracic [79, 256, 257] or Peritoneal lavage [79, 258]	Adds ~1–2 °C hr^-1^	Not commonly used unless patient is unstable and ECLS rewarming is not available.	Potential for haemorrhage, lung or bowel trauma, fluid & electrolyte shifts. Thoracic lavage has the potential to impair CPR quality.
Continuous venovenous haemofiltration [190, 242, 259–261]	Adds ~1.5–3 °C hr^-1^	Not commonly used unless ECLS rewarming not available. Requires adequate blood pressure. Heparinisation required.	Problems rare. Local vascular complications. Air embolism. Hypotension.
Haemodialysis [242, 262–266]	Adds ~2–3 °C hr^-1^	Not commonly used, patient must be able to increase cardiac output to perfuse the external circuit. Heparinisation required.	Potential for hypotension, haemorrhage, thrombosis, haemolysis, etc.
Veno-venous rewarming (usually with an ECMO circuit) [248]	~4–10 °C hr^-1^	Not commonly used. Provides no circulatory or ventilatory support in case of cardiac arrest. Patient must be able to increase cardiac output to provide circuit perfusion.	Potential for hypotension, haemorrhage, thrombosis, haemolysis, etc.
Extra-corporeal life support (VA-ECMO or CPB)	~4–10 °C hr^-1^	Preferred rewarming method for patients in cardiac arrest. CPB can use femoral route avoiding need for sternotomy [1, 42]	Potential for haemorrhage, thrombosis, haemolysis, etc. (as with all intravascular devices).

### Interpreting arterial blood gases in severe hypothermia

Acid-base disturbances in hypothermia are complex because of changes in respiration, metabolic rate, plasma solubility of CO_2_ and O_2_ and buffering capability of the blood [79, 198–200]. Initial cooling may be accompanied by hyperventilation and a respiratory alkalosis but subsequently (e.g. ≤35 °C), a mixed acidosis often ensues from a combination of falling metabolic rate and CO_2_ production [198], respiratory depression (partly to maintain CO_2_ constant in relation to metabolic production [199], increased lactate from shivering and reduced tissue perfusion, and impaired hepatic function [79]. In practice, some patients will be acidotic and others alkalotic [201], reflecting the combined-effects of concurrent pathology, individual variation and factors associated with the patient’s hypothermic events.

Blood gas samples are analysed at 37 °C, but with mathematical correction it is possible to express the results at actual body temperature. The difference has practical significance because altering the temperature changes the interpretation of the results and subsequent treatment [198]. For example, normocapnia at 37 °C will become hypocapnia at 25 °C [198, 202]. One approach assumes that 7.42 is the ideal pH at all temperatures so that management is directed towards maintaining the arterial pH at that level (pH-stat strategy). A better approach in accidental hypothermia appears to be to recognise that pH and PaCO_2_ do alter with temperature (alpha-stat strategy) [200, 203]. This approach seems to be widely accepted now in induced hypothermia, e.g. DHCA for cardiovascular surgery, and is easier to interpret because clinicians are familiar with interpreting arterial blood gases at 37 °C. Evidence extrapolated from studies in therapeutic hypothermia suggests that it is generally associated with improved cerebral perfusion and neurological outcome [198, 204–206]. In hypothermia, more oxygen will be dissolved in the blood and PaO_2_ falls [198, 207]. PaO_2_ will increase when the blood sample is rewarmed to 37 °C. To maintain the body PaO_2_ in the normal range, PaO_2_ should be corrected for current body temperature in hypothermic patients [198, 208]. Other physiological changes in hypothermia are listed in Table 4.

**Table 4:** Main physiological effects of severe hypothermia

System	Parameter	Clinical implications
CARDIOVASCULAR [79, 230, 267]	• Initial vasoconstriction (effect blocked by ethanol). Vasoconstriction fails <24 °C [268].	• Failed vasoconstriction means the patient becomes poikilothermic i.e. dependent on ambient temperature.
	• Cardiac conduction is affected by cold and changes in pH and PaO_2_ [79]. Initial tachycardia due to shivering [79] subsides as temperature drops due to decreased spontaneous depolarization of pacemaker cells leading to linear fall in pulse rate (~50 % at 28 °C) [79]. Any ECG rhythm is possible. Commonly at <32 °C, sinus bradycardia, prolonged QTc. J waves (not pathognomonic for hypothermia) best seen in leads I & V6 [79, 269–272]. Likelihood of VF is high <28 °C [267].	• Bradycardia is atropine unresponsive [79]. • A “relative” tachycardia inconsistent with patient’s temperature means something else is going on e.g. occult trauma. • Be prepared for any rhythm but expect it to be resistant to treatment until the heart rewarms. • Normal rhythm resumes on rewarming.
	• Cardiac output falls to 45 % at 25 °C [79].	• Hypotension is the norm.
	• After rewarming, mean arterial pressure, contractility, and cardiac output are decreased, especially if alcohol ingested before cooling [273].	• More prolonged depression of cardiac function after rewarming
CENTRAL NERVOUS SYSTEM	• Reflexes become increasingly sluggish as body temperature falls and become absent ≈ 28–30 °C [230, 274]. • Pupils become dilated and cease reacting to light at ≈ 28 °C [230]. • EEG shows burst suppression ≈ 22 °C and becomes isoelectric ≈ 18–20 °C [79, 275].	• The level of consciousness should be consistent with the core temperature. A significant discrepancy suggests an alternative diagnosis. • All the effects of hypothermia make it very hard to diagnose death by the usual criteria while the patient is still cold
RESPIRATORY	• Tidal volume, respiratory rate, pulmonary compliance and thoracic elasticity decrease [230]. The respiratory rate may only be five breaths per minute when the body temperature is <30 °C [79]. Sensitivity to CO_2_ is attenuated, although the hypoxic drive is maintained to deeper levels of hypothermia [230]. Cough reflex is obtunded, ciliary activity is reduced and secretions are more viscous.	• An irregular respiratory pattern can be mistaken for agonal breathing leading to premature institution of CPR. • The likelihood of a chest infection is increased.
	• Oxygen consumption and carbon dioxide production fall by about 50 % at 30 °C [230]	• Reduced CO_2_ production means it is easy to inadvertently hyperventilate hypothermic patients. Hyperoxia is also possible.
RENAL & METABOLIC	• Cold diuresis, partly due to the relative central hypervolaemia resulting from peripheral vasoconstriction [79], but also from a reduction in ADH release and resistance to its effects [230]. Alcohol will further increase the diuresis.	• Severely hypothermic patients are dehydrated. This becomes particularly important during rewarming as the consequent opening up of the peripheral circulation will lead to a rapid fall in BP.
	• Hyperglycaemia is common due to catecholamine-induced glycogenolysis, decreased insulin release and inhibition of insulin transport [79, 267].	• Hyperglycaemia can exacerbate the diuresis.
	• Glomerular filtration rate falls as cardiac output and hence renal blood flow fall [230]. At low temperatures, tubular capacity for H^+^ secretion is reduced, and hence there is a renal component of the acidosis [230].	• This makes the interpretation of acid-base more complex.
	• Hypokalaemia commonly occurs with hypothermia [230].	• If potassium replacement is given excess to the losses, hyperkalaemia may occur on rewarming [276, 277]. • Severe initial hyperkalaemia is a marker of acidosis and cell death and is therefore a sign of poor prognosis [8]
HAEMATOLOGY	• Haematocrit increases by about 2 % for every 1 °C decline in temperature [250].	• A normal haematocrit in a moderately or severely hypothermic patient suggests pre-existing anaemia or blood loss [230].
COAGULATION	• Platelet function and coagulation enzyme activity are reduced [278].	• Coagulopathy is likely and increases with decreasing core temperature. At temperatures below 33 °C coagulopathy significantly increases mortality in patients with concomitant trauma [279].

### Non-ECLS rewarming

In a hypothermic patient with CA, non-ECLS rewarming is only indicated if ECLS is not available for any reason. To be effective, rewarming by non-ECLS methods is reliant on the presence of a circulation so in a CA situation, rewarming is extremely slow, and until the heart is warm enough to restart, it is necessary to provide prolonged continuous CPR, which is very demanding. No evidence exists to guide the non-ECLS rewarming of hypothermic CA. Until an effective circulation is re-established, some experts recommend shielding the head from external heat sources (such as warming blankets) to prevent the brain temperature from rising too quickly. Regarding the choice of heating modalities, each device should be considered for its ability to assist with heat delivery against the potential to impair circulation. For example the use of pleural lavage has the potential to impair chest compression quality and may not be indicated if sufficient heat delivery can be achieved through other means. Extracorporeal devices that do not support circulation (e.g. haemodialysis) are relatively contraindicated because they can negatively impact the circulation and are relatively ineffective in the absence of native circulation to perfuse the external circuitry. The optimal rewarming rate is unknown. Theoretically, the most ‘dangerous’ time for the patient is from when the brain temperature rises >28 °C until ROSC is achieved. Current expert opinion suggests performing high-quality mechanical or manual CPR; rewarming as quickly as possible until ROSC is achieved; one or more external heat delivery devices applied only around the trunk to reduce the likelihood of afterdrop through peripheral vasodilation (e.g. heating blanket under the patient plus one or two heating blankets on the patient); warm bladder lavage through a 3-way catheter or if available, warm peritoneal lavage. Intravenous infusions should be warmed. Once the core temperature rises >28 °C, attempts at defibrillation may be considered for each degree of rewarming or with any change in observed heart rhythm. Given that studies have shown that prolonged CPR does not preclude survival [194], and that high quality CPR is possible with a mechanical device, transfer to an ECLS centre is recommended.

### Extracorporeal life support

ECLS using veno-arterial extracorporeal membrane oxygenation (VA-ECMO) or cardiopulmonary bypass (CPB) are the rewarming treatments of choice and should be performed in the presence of primary hypothermic CA or severe circulatory instability refractory to ALS due to HT III–IV. ECLS is safe and survival rates are higher than rewarming by other methods. ECLS immediately restores the circulation, maintains tissue oxygenation and CO_2_ removal and provides fast and controllable rewarming. Reported survival rates are variable (23–100 %) reflecting the wide variety of factors including environment (e.g. speed of cooling; hypoxic or non-hypoxic CA), patient factors (e.g. the presence of concurrent medical problems), causes of hypothermia (e.g. avalanche, water), issues arising during rescue, hospital selection criteria and facilities, and treatments available e.g. VA-ECMO vs. CPB [2, 6, 41, 209, 210].

The majority of patients with primary hypothermia will maintain a perfusing rhythm until <28 °C. Therefore, the indication for ECLS in arrested patients with core temperature 28–32 °C is more controversial since a larger proportion of these patients will have suffered CA from other causes and the chances of a good neurological outcome are reduced. The use of ECLS for HT III (<28 °C) patients (not in CA) may be considered in the following situations [2] (1) failure to improve with external active and minimally invasive rewarming methods, as described above (Table 3) (2) life-threatening arrhythmia; (3) hypotension (systolic blood pressure <90 mmHg); (4) respiratory failure; (5) refractory acidosis. Older patients, or those with comorbidities, that limit their tolerance for the low-flow state of HT III, may have better outcomes when managed with ECLS [209]. Young healthy HT III patients should initially be rewarmed by active external methods and minimally invasive rewarming. For patients at risk of CA (i.e. core body temperature <28 °C, ventricular arrhythmia, systolic blood pressure <90 mmHg), rewarming should ideally take place in an ECLS centre with the equipment and personnel available on site until the patient has stabilized.

In the past, most ECLS rewarming was performed using CPB [211]. More recently, VA-ECMO has become the preferred method due to its rapid availability, lower heparinisation requirements and the possibility of prolonging cardiorespiratory support if required after rewarming e.g. continuing cardiac instability, arrhythmias and post-rewarming severe pulmonary oedema [6, 11, 212]. In hypothermic CA victims, similar ROSC rates after CPB or VA-ECMO rewarming are reported though one retrospective study reported better survival with VA-ECMO [6]. Multi-organ failure is not unusual and may require prolonged VA-ECMO to maintain adequate perfusion and oxygenation until recovery of organ function [6, 213, 214]. Veno-venous ECMO is ineffective in circulatory arrest, but it can be used in a haemodynamically-stable patient with respiratory failure after rewarming with VA-ECMO.

Cannulation of the femoral artery and vein is the quickest and easiest way to establish emergency access. Sternotomy is less desirable since it is time-consuming and CPR has to be interrupted. Ideally CPR should continue until ECLS-rewarming has started [161]. Depending on the available type of rewarming (CPB vs. VA-ECMO), patients should be heparinised according to local ECLS protocols. In hypothermic multi-trauma patients, using heparinised ECLS systems and a reduction of the systemic heparinisation should be considered. New completely-heparinised VA-ECMO systems may be used for up to one week with minimal heparinisation. This makes ECLS rewarming also suitable for hypothermic CA patients with trauma and high risk of haemorrhage.

General anaesthesia should be provided to prevent the patient from waking or being aware during the procedure. It is generally advisable to start ECLS-rewarming with circuit temperatures approximately the same as the admission temperature of the patient, the idea being to avoid a large temperature gradient when ECLS commences [215]. Flows are increased gradually in an attempt to avoid the risk of gas bubble formation and ischaemia/reperfusion-induced cell damage. Gradually increasing flow to 2.2–2.5 L min^-1^m^-2^, a pressure >45 mmHg, a rewarming rate of 1 °C per 10 min, maintaining a temperature gradient 5–10 °C between the venous blood and the heat exchanger avoids gas emboli and seems safe. Rewarming rates between 1 °C 5 min^-1^ and 1 °C hour^-1^ are commonly used, but the optimal rate is unknown and thus not standardized. When using femoral access, the presence of native cardiac function will provide a counter flow in the ascending aorta and aortic arch. Ventilation must be started as soon as ECLS has been established to avoid perfusing the heart and the brain with deoxygenated blood [216–219].

ECLS should be continued until the patient has a stable cardiac rhythm, adequate native perfusion and oxygenation, and a core temperature >32 °C. Inotropes or vasopressors may be used for weaning. Targeted temperature management should be performed according to local protocols and post-resuscitation hyperthermia should be avoided [41, 220, 221]. However, the main goal is to optimize haemodynamic status and ensure adequate cerebral perfusion. Cardiac stunning or multi-organ failure are not unusual following prolonged CPR, ischaemia and subsequent ECLS reperfusion, and may require post-resuscitation VA-ECMO until adequate cardiorespiratory recovery. In a recent study, severely hypothermic patients (with and without CA) rewarmed with VA-ECMO, bi-ventricular diastolic dysfunction persisted despite systolic function recovery [222].

Termination of ECLS is considered if there is no ROSC at 32–35 °C [10, 223]. The decision to stop treatment may also be based on additional clinical information, such as uncontrollable haemorrhage, new information relating to the cause of CA or signs of severe anoxic brain injury.

Prolonged HT III and IV from any cause are relatively rare, and although premature death due to CA may occur, successful resuscitation by ECLS rewarming is possible. The outcomes of all such cases are under-reported, yet much could be learned from them. Therefore, the International Hypothermia Registry [224] was created at the University Hospital of Geneva, Switzerland, to collect case reports from across the world. If enough data can be gathered, subsequent peer-review analysis will permit the establishment of new consensus guidelines for the treatment of accidental hypothermia. All centres dealing with accidental hypothermia patients can contribute to the Registry.

### Hypothermia in children

Cooling in children is discussed briefly in the section on hypothermia and drowning. The stages, symptoms and signs of hypothermia in children are broadly similar to adults. The most important clue to significant hypothermia in children is altered mental status. The presence or absence of shivering is not a reliable marker of severity of hypothermia [46]. Unlike adults, small children may still have signs of life with a core temperature in the mid-teens [192] and a normal cardiac rhythm may resume when the temperature is 20 °C or less [192, 225]. Rewarming may be faster in children compared to adults given the larger surface-to-mass ratio and should follow the same principles as for adults [131, 133, 192, 212, 226].

### Does a country benefit from an accidental hypothermia algorithm?

In contrast to robust recommendations for the out-of-hospital management of hypothermic patients [10, 18, 227] algorithms for the in-hospital treatment are rare: According to an expert meeting in Bern, Switzerland (2013), strategies for the assessment and therapy of hypothermic patients vary widely and although several hospitals have developed algorithms, they face challenges with validation, implementation and publication of suggested guidelines [112, 228, 229]. Because hypothermic patients are not only admitted to level I hospitals but also to smaller hospitals, recommendations should focus on a small set of universally-available transport and treatment options. Accidental Hypothermia centres and treatment algorithms should be developed within the present structures of specialized departments capable of ECLS rewarming [112, 228, 230]. These algorithms include training of emergency medical services for recognition and treatment of severe hypothermia, the special requirements of hypothermic CA, pre-hospital core temperature measurement, insulation, rewarming, adequate hospital-selection for patients potentially requiring ECLS-rewarming, as described above [10, 231].

A good model for co-ordinating hypothermia care has been created in south-east Poland. Medical personnel in- and out-of-hospital have been trained in diagnosis and management of hypothermia, and ECLS rewarming facilities and intensive care treatment are available. A coordinator, who is an accidental hypothermia specialist, is available 24/7 to assist in case a critically hypothermic patient is reported [160, 229].

## Conclusions

The hospital use of minimally-invasive rewarming for non-arrested, otherwise healthy, patients with primary hypothermia and stable vital signs has the potential to substantially decrease morbidity and mortality for these patients. ECLS has revolutionised the management of hypothermic CA, with survival rates approaching 100 % in some cases. Hypothermic patients with risk factors for imminent CA (e.g. temperature <28 °C, ventricular arrhythmia, systolic blood pressure <90 mmHg), and those who have already arrested, should be transferred directly to an ECLS-centre. Cardiac arrest patients must receive CPR during transfer. In difficult conditions mechanical or intermittent CPR should be considered. ECLS has substantially improved survival and is the treatment of choice in the patient with unstable circulation or CA. Modern post-resuscitation care should be implemented following hypothermic arrest. Structured protocols should be in place to optimise pre-hospital triage, transport and treatment as well as in-hospital management, including detailed criteria and protocols for the use of ECLS and post-resuscitation care.

Based on new evidence, additional clinical experience with ECLS rewarming and clearer management guidelines and documentation, the treatment of accidental hypothermia has been refined.

## Abbreviations

ALS: Advanced life support
CA: Cardiac arrest
CO_2_: Carbon dioxide
CPB: Cardiopulmonary bypass
CPC: Cerebral performance category
CPR: Cardiopulmonary resuscitation
DHCA: Deep hypothermic circulatory arrest
ECLS: Extracorporeal life support
ECMO: Extracorporeal membrane oxygenation
ETCO_2_: End tidal carbon dioxide
HT: Hypothermia
NIRS: Near infrared spectroscopy
O_2_: Oxygen
PaO_2_: Partial arterial oxygen pressure
ROSC: Return of spontaneous circulation
rSO_2_: Regional oxygen saturation
VA: Veno-arterial
